# The Musculoskeletal Tumor Society Scoring system is a valid subjective and objective tool to evaluate outcomes of surgical treatment of patients affected by upper and lower extremity tumors

**DOI:** 10.1007/s12306-024-00815-3

**Published:** 2024-03-14

**Authors:** A. Rizzo, M. Paderno, M. F. Saccomanno, F. Milano, G. Milano

**Affiliations:** 1 Department of Medical and Surgical Specialties, Radiological Sciences, and Public Health, University of Brescia, Brescia, Italy; 2 Department of Bone and Joint Surgery, Spedali Civili, Brescia, Italy; 3 Department of Psychology, University of Milan-Bicocca, Milan, Italy

**Keywords:** Upper limb tumors, Lower limb tumors, Patient reported outcome measures, Functional evaluation

## Abstract

**Purpose:**

The main purpose of the present study was to evaluate if there is a difference between objective or subjective administration of the MSTS score in a cohort of patients affected by musculoskeletal oncological diseases.

**Materials and methods:**

All patients who underwent surgery for bone or soft tissue localization of neoplastic disease in lower or upper limb from June 2015 to June 2020 were considered eligible. In order to administer the score as a PROM, the MSTS was first translated and cross-culturally adapted in Italian. During follow up visits, all patients filled out Italian versions of SF36, TESS and MSTS. Psychometric properties of the Italian version of MSTS were analyzed. Correlation between objective and self-administered MSTS score was assessed through Pearson’s coefficient.

**Results:**

A finale sample of 110 patients were included: 59 affected by lower extremity involvement and 51 affected by upper extremity involvement. The Italian version of the MSTS score showed good psychometric properties for both lower and upper extremity. The correlation between self-administered and hetero-administered version of the questionnaire was as high as *r* = 0.97 for lower extremities and *r* = 0.96 for upper extremities.

**Conclusions:**

The Italian version of the MSTS is a valid tool to evaluate outcomes of surgical treatment of patients affected by extremities tumors and it can be used as a subjective tool for both lower and upper extremity.

## Introduction

Treatments of oncological diseases of musculoskeletal system mostly aim to guarantee function and quality of life (QoL) at their best, especially when eradication of the neoplasia is not achievable. Although in the past most outcome studies focused on survival rate and local recurrences as primary outcomes, in the last decades more emphasis has been placed on patient’s perspective. It has been shown that taking into account patient perception promotes communication, improves decision-making process and increases patient satisfaction. Objective measures integrated with patient perception could provide better medical care [1–3].

The Musculoskeletal Tumor Society (MSTS) score was developed in 1993 as an objective tool to measure functional outcome in patients affected by neoplasms [4]. Even if the MSTS score has been never properly validated in its original version [4], it is widely used in clinical practice. As a matter of fact, the original version underwent cross-cultural adaptation and validation in several languages, such as Greek [5], Danish [6], Brazilian [7], Chinese [8], Japanese [9], French [10] and Turkish [11].

The score is available for upper and lower limb [12]. Main strengths of the score mainly rely on ease of use and briefness [13]. Main concern is that the MSTS score was formulated as an objective tool (hetero-administered), but it is currently worldwide used as a patient-reported outcome measure (PROM) (self-administered).

The main purpose of the present study is to evaluate if there is a difference between objective or subjective administration of the MSTS score in a cohort of patients affected by musculoskeletal oncological diseases. The hypothesis of the study is that there are no differences between patient- and clinician-reported outcomes using the MSTS score for both lower and upper limb.

## Materials and methods

### Study design

An observational study was conducted, after approval of the study protocol by the local ethic committee (NP 4912 Spedali Civili, Brescia).

### Patients

All patients who underwent surgery for bone or soft tissue localization of neoplastic disease in lower or upper limb from June 2015 to June 2020 at Spedali Civili in Brescia, Italy, were considered eligible for the study. Patients were included regardless of previous treatment and disease stage. Onco-emathologic diseases were also included. Inclusion criteria also included: Italian as mother language, age of 18 years or above, minimum twelve-month follow-up from surgery, willingness to enter the study and ability to provide informed consent. Patients with a Karnofsky’s score lower than 30% [14], those who did not undergo surgical treatment and those who had diagnosis of dementia (any type) or were in a state of altered metal status were excluded.

### Intervention

In order to administer the score as a PROM, the first part of the study consisted of translation and cross-cultural adaptation of an Italian version of the questionnaire according to well-established guidelines [15, 16]. Questionnaires were then administered during the postoperative follow-up visits in an outpatient setting. Thirty to sixty minutes after completion of self-administered questionnaires, patients underwent an interview by an orthopedic surgeon, based on the MSTS questionnaire. The examiner was blinded to the patients’ answers at the self-administration of the questionnaire. Retest was conducted after a period of two weeks after first administration in order to avoid any recall bias.

### Outcome measures

Besides the Italian version of MSTS questionnaire, all patients filled out the national validated version of the Toronto Extremity Savage Score (TESS) [17]. Each patient completed the upper or the lower limb version of both MSTS and TESS score, depending on the localization of the disease. The national validated version of the SF-36 [18] was used as general health measurement.

The MSTS questionnaire [4] consists of six domains, each scored on a scale from 0 to 5, with a higher score indicating better function. The total score, ranging from 0 (maximum disability) to 30 (no impairment), can be transformed to a point scale of 0 to 100.

The TESS score assesses functional outcome in musculoskeletal tumor patients aged 12–85 years [19]. It consists of 29 items for upper extremity and 30 items for lower extremity. The degree of disability is rated from 0 (complete disability) to 5 (no functional impairment) in each item. Similar to MSTS, the final TESS score can be converted to a score ranging from 0 to 100 points.

The SF-36 is a non-pathology-related questionnaire aiming to test both physical and mental components of patient perception of QoL. It is composed of 36 questions divided into eight different domains. Each of these domains can be rated from 0 (worst) to 100 (best).

### Data analysis

Sample size was estimated according to established guidelines for questionnaire validation [16, 17]. The Italian version of MSTS questionnaire was first test retested on at least 30 patients per group (upper and lower limb). Psychometric properties of the questionnaire were then assessed on a sample of 50 patients per group.

All the data were analyzed by SPSS 25 (IBM Statistics, Harmonk, NY, USA). Descriptive statistics were used to report scores and answers distribution for each question. Data normality was ascertained by Shapiro–Wilk test. Discrete data were expressed as mean ± standard deviation in case of normal data distribution, otherwise as median and interquartile range (IQR). Categories were expressed as frequencies and percentages.

Ceiling and floor effect were considered significant if more than 15% of patient reached the lowest or the highest possible score, respectively.

Content validity could not be tested through the multi-trait analysis because each question corresponds to a domain. The structure of the questionnaire was determined by the factor analysis. Factor’s number was calculated using Kaiser criteria (eigenvalue > 1) and a scree plot.

Construct validity was calculated through Pearson’s coefficient correlation. The Italian version of MSTS score was compared to TESS and SF-36 score. Correlation between objective and self-administered MSTS score was assessed through Pearson’s coefficient. Correlation was deemed as very weak when ranging 0 to 0.19, weak if between 0.20 and 0.39, mild if between 0.40 and 0.69, strong if between 0.70 and 0.89 and very strong if between 0.90 and 1 [15].

Reliability was assessed by internal consistency and test–retest reliability. Cronbach’s alpha coefficient measured internal consistency for every domain. Internal consistency higher than 0.70 indicates good reproducibility [20]. Intraclass correlation coefficient (ICC) measured test–retest reliability. ICC values ranged between 0 (absolute disagreement) and 1 (maximum disagreement). Values were interpreted as follows: poor reliability when less than 0.50, moderate when between 0.50 and 0.75, good when between 0.75 and 0.90 and excellent when greater than 0.90 [21].

Significance at probability tests was estimated for *p* value < 0.05.

## Results

No major issues were encountered during translation from the original version. No major difficulties in comprehension were revealed during testing the pre-final version. Patients took about 5–10 min to complete the questionnaire (see Appendix 1).

The psychometric properties were tested on a finale sample of 110 patients: 59 affected by lower extremity involvement and 51 affected by upper extremity involvement. Patients’ characteristics are shown in Tables 1 and 2, for upper and lower limb, respectively.

**Table 1:** Upper limb involvement: patients characteristics

Characteristic	*N* (%)
Age (years)	55 ± 16
Follow-up (months)	45 ± 21
Localization	
Shoulder	10 (20)
Arm	18 (35)
Elbow	2 (4)
Forearm	7 (14)
Wrist	5 (10)
Hand	9 (18)
Histotype	
Metastatic lesions (including multiple myeloma)	6 (12)
Sarcomas	9 (18)
Osteosarcoma	2 (4)
Chondrosarcoma	1 (2)
Liposarcoma	2 (4)
Other soft tissue sarcomas	4 (8)
Lipoma	7 (14)
Fibrolipoma	5 (10)
Enchondroma	3 (6)
Schwannoma	5 (10)
Angiolipoma	2 (4)
Well differentiated liposarcoma	1 (2)
Cyst	1 (2)
Giant cell tumor	8 (16)
Neurofibroma	1 (2)
Nodular fasciitis	1 (2)
Glomic tumor	1 (2)
Nora’s disease	1 (2)
Karnofsky’s score	
100	32 (63)
90	11 (22)
80	5 (10)
70	3 (6)
< 70	0
Surgical treatment	
Amputation	0
Internal fixation	4 (8)
Prosthetic reconstruction	3 (6)
Wide resection	44 (86)
Medical treatment	
No systemic therapy	41 (80)
Neoadiuvant systemic therapies	5 (10)
Adiuvant systemic therapies	3 (6)
Both neoadiuvant and adiuvant therapies	2 (4)

**Table 2:** Lower limb involvement: patients characteristics

Characteristic	*N* (%)
Age (years)	52 ± 17
Follow-up (months)	40 ± 20
Localization	
Hip	9 (17)
Thight	24 (41)
Knee	7 (12)
Leg	12 (20)
Ankle	1 (2)
Foot	6 (10)
Histotype	
Metastatic lesions (including multiple myeloma)	10 (17)
Sarcomas	17 (29)
Osteosarcoma	3 (5)
Chondrosarcoma	1 (2)
Liposarcoma	5 (8)
Other soft tissue sarcomas	8 (14)
Lipoma	8 (14)
Fibrolipoma	1 (2)
Mixoma	2 (3)
Schwannoma	3 (5)
Exostosis	2 (3)
Well differentiated liposarcoma	1 (2)
Cyst	6 (10)
Giant cell tumor	2 (3)
Hemangioma	3 (5)
Osteoid osteoma	1 (2)
Chondroma	1 (2)
Villonodular synovites	1 (2)
Mixed tumor	1 (2)
Karnofsky’s score	
100	36 (61)
90	11(19)
80	6 (10)
70	6 (10)
< 70	0
Surgical treatment	
Amputation	2 (3)
Internal fixation	3 (5)
Prosthetic reconstruction	9 (15)
Wide resection	45 (76)
Medical treatment	
No systemic therapy	41 (69)
Neoadiuvant systemic therapies	7 (12)
Adiuvant systemic therapies	7 (12)
Both neoadiuvant and adiuvant therapies	4 (7)

### Psycometric properties of MSTS score for upper extremity

The descriptive statistics data are shown in Tables 3 (self-administered) and 4 (hetero-administered). No missing data were reported, thus confirming that the translated questionnaire was well understood by the patients. A ceiling effect > 15% was observed for all items in both administration modalities.

**Table 3:** MSTS upper extremity (self-administered)

Scale	Missing (%)	Observed values						
		Mean	SD	Lowest	Highest	Range	% at floor	% at ceiling
Pain (0–100)	0	84.7	25.5	20	100	80	0	62.7
Function (0–100)	0	85.1	26.5	20	100	80	0	68.6
Emotional (0–100)	0	83.1	30.8	0	100	100	5.9	72.5
Hand position (0–100)	0	88.6	19.7	20	100	80	0	68.6
Manual dexterity (0–100)	0	91.8	14.5	40	100	60	0	70.6
Lifting ability (0–100)	0	84.7	19.4	20	100	80	0	52.9

Descriptive statistics for scales (normalized scores)

**Table 4:** MSTS upper extremity (hetero-administered)

Scale	Missing (%)	Observed values						
		Mean	SD	Lowest	Highest	Range	% at floor	% at ceiling
Pain (0–100)	0	86.3	22.8	0	100	100	2	62.7
Function (0–100)	0	85.5	22.3	20	100	80	0	62.7
Emotional (0–100)	0	89	23.1	0	100	100	3.9	72.5
Hand position (0–100)	0	89.8	16.2	40	100	60	0	64.7
Manual dexterity (0–100)	0	91.4	16.1	20	100	80	0	70.6
Scale	0	84.3	23.8	0	100	100	2	56.9

Descriptive statistics for scales (normalized scores)

**Fig. 1:**
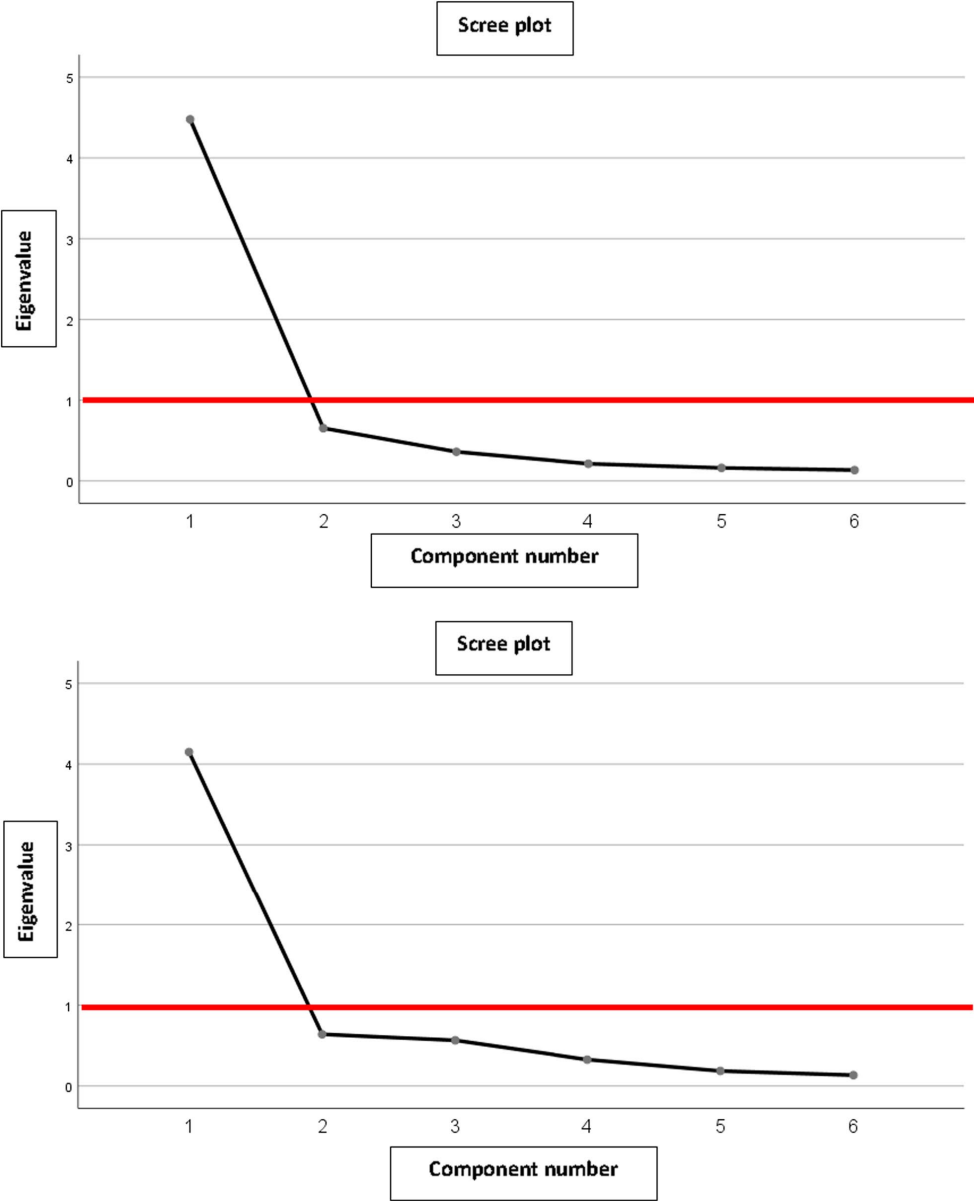
**A** MSTS upper extremity (self-administered). Scree plot for factor analysis. **B** MSTS upper extremity (hetero-administered). Scree plot for factor analysis

Factor analysis, as indicated in the scree plots (Fig. 1), showed that the appropriate number of factors was 1. This was visible in both the self-administered and hetero-administered modalities.

The assessment of construct validity (Appendix 2) showed that both administration modalities had overall good correlation with TESS (*r* = 0.78 and *r* = 0.80 for the self-administered version and for the hetero-administered version, respectively). On the opposite, both administrations showed poor correlation with SF-36: (*r* = 0.19 and and *r* = 0.1 for the self-administered version and for the hetero-administered version, respectively).

Internal consistency was as high as Cronbach’s alpha = 0.84 for self-admnistered MSTS and 0.77 for hetero-administered MST versions (Appendix 3). Test–retest reliability was good in both versions, with an overall ICC of 0.84 for self-administered version and 0.78 for hetero-administered version (Appendix 4).

Correlation between self-administered and hetero-administered version of the questionnaire was high (*r* = 0.96) (Table 5).

**Table 5:** MSTS Upper extremity

Items	E—Pain		E—Function		E—Emotional		E—Hand position		E—Manual dexterity		E—Lifting ability		E—MSTS Overall	
	*r*	*p*	*r*	*p*	*r*	*p*	*r*	*p*	*r*	*p*	*r*	*p*	*r*	*p*
A—Pain	0.788	< 0.0001											0.751	< 0.0001
A—Function			0.859	< 0.0001									0.918	< 0.0001
A—Emotional					0.848	< 0.0001							0.841	< 0.0001
A—Hand position							0.884	< 0.0001					0.807	< 0.0001
A—Manual dexterity									0.784	< 0.0001			0.820	< 0.0001
A—Lifting ability											0.821	< 0.0001	0.803	< 0.0001
A —MSTS Overall	0.863	< 0.0001	0.860	< 0.0001	0.668	< 0.0001	0.878	< 0.0001	0.739	< 0.0001	0.792	< 0.0001	0.961	< 0.0001

Correlation between self-administered (A) and hetero-administered (E) versions of the questionnaire

### Psycometric properties of MSTS score for lower extremity

Descriptive statistics are shown in Tables 6 (self-administered) and 7 (hetero-administered). No missing data were reported. A ceiling effect > 15% was observed for all items in both administration modalities.

**Table 6:** MSTS Lower extremity (self-administered)

Scale	Missing (%)	Observed values						
		Mean	SD	Lowest	Highest	Range	% at floor	% at ceiling
Pain (0–100)	0	78.3	29.8	0	100	100	3.4	50.8
Function (0–100)	0	74.2	32.8	0	100	100	5.1	50.8
Emotional (0–100)	0	79	30.7	0	100	100	5.1	59.3
Supports (0–100)	0	84.4	32.4	0	100	100	6.8	78
Walking (0–100)	0	82	27.2	0	100	100	3.4	57.6
Gait (0–100)	0	81	30.7	0	100	100	5.1	62.7

Descriptive statistics for scales (normalized scores)

**Table 7:** MSTS Lower extremity (hetero-administered)

Scale	Missing (%)	Observed values						
		Mean	SD	Lowest	Highest	Range	% at floor	% at ceiling
Pain (0–100)	0	81.7	24.2	0	100	100	1.7	50.8
Function (0–100)	0	78.6	26.2	0	100	100	3.4	47.5
Emotional (0–100)	0	82.7	28.2	0	100	100	5.1	64.4
Supports (0–100)	0	86.1	31.8	0	100	100	6.8	81.4
Walking (0–100)	0	84.1	26.2	0	100	100	3.4	64.4
Gait (0–100)	0	80.7	28	0	100	100	3.4	57.6

Descriptive statistics for scales (normalized scores)

**Fig. 2:**
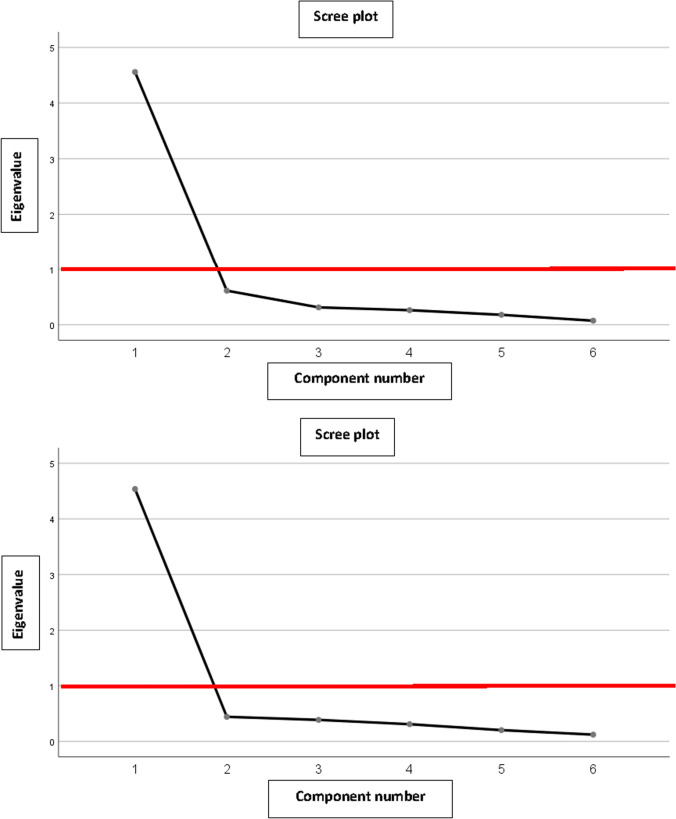
**A** MSTS lower extremity (self-administered). Scree plot for factor analysis. **B** MSTS lower extremity (hetero-administered). Scree plot for factor analysis

Factor analysis, as indicated in the scree plots (Fig. 2**)**, showed that the appropriate number of factors is 1. This was confirmed for both the self-administered and the hetero-administered modalities.

Assessment of construct validity showed that both administration modalities had overall strong correlation with TESS, as high as *r* = 0.80 for both versions, and mild correlation with SF-36, equal to *r* = 0.5 for both versions (Appendix 5).

Internal consistency was very high for both self- and hetero-administered MSTS versions. Self-administered version showed an overall Cronbach’s alpha = 0.96, while the hetero-administered version reached an overall Cronbach’s alpha = 0.98 (Appendix 6). Test–retest reliability was excellent in both versions: ICC = 0.96 for the self-administered version and ICC = 0.98 for the hetero-administered version (Appendix 7).

The correlation between self-administered and hetero-administered version of the questionnaire was as high as *r* = 0.97 (Table 8).

**Table 8:** MSTS Lower extremity. Correlation between self-administered (A) and hetero-administered (E) versions of the questionnaire

Items	E—Pain		E—Function		E—Emotional		E—Supports		E—Walking		E—Gait		E—MSTS Overall	
	*r*	*p*	*r*	*p*	*r*	*p*	*r*	*p*	*r*	*p*	*r*	*p*	*r*	*p*
A—Pain	0.877	< 0.0001											0.793	< 0.0001
A—Function			0.832	< 0.0001									0.814	< 0.0001
A—Emotional					0.880	< 0.0001							0.782	< 0.0001
A—Supports							0.971	< 0.0001					0.887	< 0.0001
A—Walking									0.907	< 0.0001			0.863	< 0.0001
A—Gait											0.928	< 0.0001	0.922	< 0.0001
A —MSTS Overall	0.818	< 0.0001	0.887	< 0.0001	0.804	< 0.0001	0.834	< 0.0001	0.822	< 0.0001	0.881	< 0.0001	0.968	< 0.0001

## Discussion

Main finding of the present study was that MSTS score can be interchangeably used as a PROM or as an objective tool because both administration modalities showed to be valid and the correlation between the two was very high. At the same time, it must be highlighted that the Italian version of the MSTS score showed good psychometric properties for both lower and upper extremity.

MSTS score has been originally developed as a clinician-administered questionnaire and it is now widely used to evaluate residual function in patients with extremity tumors [4]. However, even if it has been developed to be completed by an examiner, MSTS score is often reported in the literature as a self-administered tool [22] or even as a mixed version with some questions completed by the patient and some others completed by the clinician.

Marchese et al. [23] and Ginsberg et al. [24] assessed functional outcomes in patients affected by lower extremity sarcomas. Both studies used the MSTS score by asking patients to complete pain, emotional acceptance and supports, while physical therapists rated gait and walking abilities.

Janssen et al. [25] first compared self- and hetero-administered modality of the original version, in a study about functional outcome after surgery in patients affected by lower and upper extremity bone metastasis. According to the authors, clinician reports overestimate function as compared to the patient perceived score. This assumption strongly differs from the outcome of the present study. The reason probably relies in the study design. As recognized by the authors [25], they collected the clinician reports by resuming information from previously noted medical records, but the score was developed to be completed at the time of consultation. As a matter of fact, the discrepancy was the largest for the common overall function and emotional acceptance domains. In the present study, patients were directly visited and interviewed by the clinician, who filled out the form during the clinical examination, thus reducing the risk of possible misinterpretations.

Looking at the results of the psychometric properties, some issues deserve further explanation.

The original version of MSTS score was never properly validated, therefore results of the present study can be only compared to other cross-cultural adaptations [5–11, 26]. We observed that ceiling effect was high for all questions both for upper and lower extremity forms, which it means that the questionnaire cannot distinguish higher functioning patients. This finding is in agreement with previous studies [6, 7, 26, 27]. At least two possible explanations can be attempted. First, MSTS score was developed in a time when limb savage surgery and reconstructive options were less common, and expectations on functional results were quite low. Secondly, a sensitivity analysis aiming to distinguish between hystotypes or at least between aggressive, intermediate and benign tumors could have probably lowered or partially better explained the effect. Saebye et al. [6], in their study on cross-cultural adaptation and validation of Danish version of MSTS score, found no ceiling effect among patients with lower extremity bone sarcomas or high aggressive tumors after stratification. Rebolledo et al. [7] provided cross-cultural adaptation and validation of the Brazilian Portuguese MSTS score. They only included patients who underwent limb salvage surgery or amputation for primary sarcoma of the lower limb, and no ceiling effect was reported. Unfortunately, sample size of the present study did not allow stratification for hystotype, albeit a strong and valid outcome measurement tool should be as universal as possible. Inclusion criteria of the present study were kept wide on purpose. In fact, MSTS score is widely used for any kind of tumor, and therefore, it was deemed important to be as inclusive as possible.

In agreement with previous translations of MSTS questionnaire [5, 7–9, 26], we observed that the instrument composed by all six items is able to evaluate one latent factor (e.g., lower or upper limb function).

TESS and SF-36 were chosen to test the construct validity to be consistent with the previously translated versions [5, 7–9, 11, 26]. MSTS and TESS reported moderate to strong correlation in all studies. Results differed and became controversial when it comes to SF-36. While some studies [9, 11, 26, 27] showed better correlation with the physical component of SF-36, some others did not [5]. However, the SF-36 is a tool designed and widely used as a general health and health-related QoL assessment measurement, thus this controversial correlation can be easily understood.

In terms of reliability, we found a Cronbach’s alpha coefficient > 0.95 for both upper and lower extremity in each administration modality. A general accepted rule is that Cronbach’s alpha of 0.6–0.7 indicates an acceptable level of reliability, and 0.8 or greater a very good level. However, values higher than 0.95 are not necessarily good, since they might be an indication of redundance [28]. Overall, previous cross-cultural adaptations of MSTS score showed a Cronbach’s alpha between 0.70 and 0.90. Once again, the different values of Cronbach’s alpha in the present study are possibly due to indirect influence from external factors such as heterogeneity of study population [20].

The present study has some limitations. First, as already mentioned, a sensitivity analysis could have been clarified some controversial psychometric properties. However, it must be highlighted that results are comparable to other cross-cultural adaptations of the questionnaire. Therefore, the major flaw is probably that the original version has never been properly tested. At the same time, as the MSTS score is the most popular and widely used, it was mandatory to provide an Italian version. The added value of the study relies on its main purpose: the comparison between self- and hetero-administration of the score. Second, only patients attending the outpatient clinic were asked to participate in the study. It must be considered that somehow patients with progressive disease or unsatisfied patients are less likely to be available for testing in the same setting.

In conclusion, the Italian version of the MSTS is a valid tool to evaluate outcomes of surgical treatment of patients affected by extremities tumors and it can be used as a subjective tool for both lower and upper extremity.

## Appendix 1

See appendix Tables

**Table 9:** Italian version of MSTS score for upper extremity

Punteggio	Dolore	Funzione	Soddisfazione	Mobilità dell’arto superiore	Abilità manuali	Abilità di sollevamento di pesi
5	Nessun dolore	Non ridotta	Molto soddisfatto	Non limitazioni al movimento	Nessuna limitazione	Normale tolleranza al carico
4	Intermedio	Intermedia	Intermedia	Intermedio	Intermedia	Intermedio
3	Modesto—non invalidante	Ridotta per attività ricreative	Soddisfatto	Incapacità di sollevare l'arto sopra le spalle – impossibilità di svolgere prono supinazione	Perdita dei movimenti fini	Limitazioni
2	Intermedio	Intermedia	Intermedia	intermedio	intermedio	Intermedio
1	Moderato—talvolta invalidante	Parzialmente ridotta per attività occupazionali	Accettazione	Incapacità di sollevare l'arto sopra il punto vita	Perdita dei movimenti di pinza	Solo con aiuto
0	Severo—Persistentemente invalidante	Totale impossibilità di svolgere attività occupazionale	Insoddisfatto	Incapacità di sollevare l'arto	Perdita dei movimenti di presa	Incapacità

9 and

**Table 10:** Italian version of MSTS score for lower extremity

Punteggio	Dolore	Funzione	Soddisfazione	Uso di ausili	Tolleranza alla deambulazione	Andatura
5	Nessun dolore	Non ridotta	Molto soddisfatto	Non limitazioni alla deambulazione senza ausili	Nessuna limitazione	Normale
4	Intermedio	Intermedia	Intermedia	Intermedio	Intermedia	Intermedio
3	Modesto—non invalidante	Ridotta per attività ricreative	Soddisfatto	Uso di una stampella	Limitata a brevi distanze	Zoppia lieve
2	Intermedio	intermedia	Intermedia	Intermedio	intermedio	Intermedio
1	Moderato—talvolta invalidante	Parzialmente ridotta per attività occupazionali	Accettazione	Uso di un bastone canadese	Brevi spostamenti in ambienti chiusi	Zoppia importante
0	Severo—Persistentemente invalidante	Totale impossibilità di svolgere attività occupazionale	Insoddisfatto	Uso di due bastoni canadesi	Deambulazione non autonoma	Zoppia che limita la deambulazione

10.

## Appendix 2

See appendix Tables

**Table 11:** Construct validity of MSTS for upper extremity (self-administered)

Scales	TESS		SF-36	
	*R*	*p*	*r*	*p*
Pain	0.553	< 0.0001	0.329	0.018
Function	0.752	< 0.0001	0.153	0.285
Emotional	0.609	< 0.0001	0.019	0.892
Hand position	0.677	< 0.0001	0.213	0.133
Manual dexterity	0.722	< 0.0001	0.207	0.145
Lifting ability	0.746	< 0.0001	0.091	0.525
MSTS Overall	0.776	< 0.0001	0.188	0.188

Correlation between MSTS and other measurement scales (TESS and SF-36)

11 and

**Table 12:** Construct validity of MSTS for upper extremity (hetero-administered)

Scales	TESS		SF-36	
	*R*	*p*	*R*	*p*
Pain	0.634	< 0.0001	0.125	0.382
Function	0.728	< 0.0001	0.072	0.614
Emotional	0.569	< 0.0001	-0.113	0.430
Hand position	0.743	< 0.0001	0.186	0.191
Manual dexterity	0.622	< 0.0001	0.108	0.450
Lifting ability	0.704	< 0.0001	0.144	0.313
MSTS Overall	0.800	< 0.0001	0.097	0.497

Correlation between MSTS and other measurement scales (TESS and SF-36)

12.

## Appendix 3

See appendix Tables

**Table 13:** Internal consistency of MSTS for upper extremity (self-administered)

Items	Pain	Function	Emotional	Hand position	Manual dexterity	Lifting ability	MSTS Overall
Pain	**0.846**	0.712	0.348	0.564	0.623	0.688	0.515
Function	0.840	**0.866**	0.407	0.777	0.660	0.895	0.622
Emotional	0.864	0.716	**0.845**	0.606	0.677	0.675	0.565
Hand position	0.656	0.849	0.351	**0.847**	0.634	0.820	0.780
Manual dexterity	0.635	0.741	0.551	0.586	**0.858**	0.770	0.798
Lifting ability	0.753	0.782	0.334	0.664	0.685	**0.878**	0.712
MSTS Overall	0.883	0.866	0.545	0.751	0.771	0.889	**0.837**

Bold values show a high internal consistency for each domain

Reliability coefficients and inter-scale correlations at test–retest evaluation

13 and

**Table 14:** Internal consistency of MSTS for upper extremity (hetero-administered)

Items	Pain	Function	Emotional	Hand position	Manual dexterity	Lifting ability	MSTS Overall
Pain	**0.935**	0.767	0.469	0.472	0.719	0.595	0.573
Function	0.755	**0.868**	0.543	0.640	0.577	0.583	0.513
Emotional	0.545	0.541	**0.952**	0.365	0.517	0.427	0.243
Hand position	0.745	0.837	0.376	**0.833**	0.527	0.746	0.766
Manual dexterity	0.517	0.516	0.292	0.171	**0.912**	0.277	0.476
Lifting ability	0.617	0.822	0.441	0.755	0.569	**0.839**	0.647
MSTS Overall	0.834	0.866	0.654	0.640	0.759	0.685	**0.772**

Bold values show a high internal consistency for each domain

Reliability coefficients and inter-scale correlations at test–retest evaluation

14.

## Appendix 4

See appendix Tables

**Table 15:** Test–retest reliability of MSTS for upper extremity (self-administered)

Scales	ICC	95% CIs	
		Lower limit	Upper limit
Pain	0.850	0.684	0.929
Function	0.870	0.725	0.938
Emotional	0.839	0.664	0.923
Hand position	0.851	0.685	0.929
Manual dexterity	0.841	0.652	0.926
Lifting ability	0.863	0.698	0.936
MSTS Overall	0.841	0.665	0.924

Intraclass correlation coefficients at test–retest evaluation

*ICC* Intraclass correlation coefficient

15 and

**Table 16:** Test–retest reliability of MSTS for upper extremity (hetero-administered)

Scales	ICC	95% CIs	
		Lower limit	Upper limit
Pain	0.933	0.861	0.968
Function	0.872	0.730	0.939
Emotional	0.951	0.897	0.976
Hand position	0.836	0.655	0.922
Manual dexterity	0.914	0.819	0.959
Lifting ability	0.836	0.659	0.922
MSTS Overall	0.777	0.530	0.894

Intraclass correlation coefficients at test–retest evaluation

*ICC* Intraclass correlation coefficient

16.

## Appendix 5

See appendix Tables

**Table 17:** Construct validity of MSTS for lower extremity (self-administered)

Scales	TESS		SF-36	
	*r*	*p*	*r*	*p*
Pain	0.586	< 0.0001	0.317	0.014
Function	0.716	< 0.0001	0.492	< 0.0001
Emotional	0.595	< 0.0001	0.331	0.010
Supports	0.698	< 0.0001	0.448	< 0.0001
Walking	0.824	< 0.0001	0.540	< 0.0001
Gait	0.763	< 0.0001	0.461	< 0.0001
MSTS Overall	0.798	< 0.0001	0.494	< 0.0001

Correlation between MSTS and other measurement scales (TESS-Lower extremity and SF-36)

17 and

**Table 18:** Construct validity of MSTS for lower extremity (hetero-administered)

Scales	TESS		SF-36	
	*r*	*p*	*r*	*p*
Pain	0.648	< 0.0001	0.409	0.001
Function	0.770	< 0.0001	0.549	< 0.0001
Emotional	0.538	< 0.0001	0.366	0.004
Supports	0.680	< 0.0001	0.448	< 0.0001
Walking	0.832	< 0.0001	0.529	< 0.0001
Gait	0.707	< 0.0001	0.523	< 0.0001
MSTS Overall	0.799	< 0.0001	0.541	< 0.0001

Correlation between MSTS and other measurement scales (TESS-Lower extremity and SF-36)

18.

## Appendix 6

See appendix Tables

**Table 19:** Internal consistency of MSTS for lower extremity (self-administered)

Items	Pain	Function	Emotional	Supports	Walking	Gait	MSTS Overall
Pain	**0.909**	0.779	0.628	0.759	0.648	0.705	0.862
Function	0.821	**0.891**	0.532	0.671	0.603	0.724	0.821
Emotional	0.581	0.531	**0.698**	0.695	0.557	0.660	0.708
Supports	0.560	0.579	0.397	**0.997**	0.764	0.831	0.829
Walking	0.512	0.742	0.622	0.736	**0.906**	0.687	0.822
Gait	0.593	0.655	0.410	0.929	0.732	**0.956**	0.848
MSTS Overall	0.745	0.774	0.589	0.911	0.782	0.860	**0.961**

Bold values show a high internal consistency for each domain

Reliability coefficients and inter-scale correlations at test–retest evaluation

19 and

**Table 20:** Internal consistency of MSTS for lower extremity (hetero-administered)

Items	Pain	Function	Emotional	Supports	Walking	Gait	MSTS Overall
Pain	**0.883**	0.849	0.440	0.661	0.659	0.662	0.836
Function	0.749	**0.919**	0.256	0.779	0.783	0.895	0.905
Emotional	0.568	0.607	**0.771**	0.600	0.552	0.556	0.721
Supports	0.543	0.634	0.285	**0.983**	0.897	0.832	0.882
Walking	0.577	0.650	0.271	0.633	**0.865**	0.661	0.743
Gait	0.685	0.839	0.460	0.809	0.762	**0.938**	0.925
MSTS Overall	0.743	0.843	0.456	0.861	0.852	0.860	**0.979**

Bold values show a high internal consistency for each domain

Reliability coefficients and inter-scale correlations at test–retest evaluation

20.

## Appendix 7

See appendix Tables

**Table 21:** Test–retest reliability of MSTS for lower extremity (self-administered)

Scales	ICC	95% Cis	
		Lower limit	Upper limit
Pain	0.911	0.816	0.957
Function	0.892	0.777	0.948
Emotional	0.704	0.380	0.858
Supports	0.997	0.995	0.999
Walking	0.907	0.808	0.955
Gait	0.956	0.909	0.979
MSTS Overall	0.962	0.921	0.982

Intraclass correlation coefficients at test–retest evaluation

*ICC* Intraclass correlation coefficient

21 and

**Table 22:** Test–retest reliability of MSTS for lower extremity (hetero-administered)

Scales	ICC	95% CIs	
		Lower limit	Upper limit
Pain	0.882	0.757	0.943
Function	0.902	0.760	0.956
Emotional	0.749	0.475	0.879
Supports	0.983	0.965	0.992
Walking	0.863	0.719	0.934
Gait	0.940	0.876	0.971
MSTS Overall	0.979	0.957	0.990

Intraclass correlation coefficients at test–retest evaluation

*ICC* Intraclass correlation coefficient

22.

## Declarations

### Location where the work was performed

Department of Bone and Joint Surgery, Spedali Civili, Brescia, Italy.
